# Simplified Loop-Mediated Isothermal Amplification-Based Method for Point-of-Care Detection of *Streptococcus pneumoniae* in Low-Resource Settings

**DOI:** 10.1021/acsomega.5c01541

**Published:** 2025-07-09

**Authors:** Petr Jeřábek, Markéta Martínková, Matúš Friček, Christa E. van der Gaast − de Jongh, Denis R. Katundu, Niels van Heerbeek, Corné H. van den Kieboom, Marien I. de Jonge, Václav Martínek

**Affiliations:** 1 Department of Biochemistry, Faculty of Science, 37740 Charles University, Prague 128 00, The Czech Republic; 2 Department of Laboratory Medicine, Laboratory of Medical Immunology, Radboud Center for Infectious Diseases, 691252 Radboud University Medical Center, Nijmegen 6525 GA, The Netherlands; 3 Department of Otolaryngology, 108094 Kilimanjaro Christian Medical University College, Kilimanjaro Tanzania; 4 Department of Otolaryngology, Head and Neck Surgery, 691252 Radboud University Medical Center, Nijmegen 6525 GA, The Netherlands; 5 Xheal B.V., Reinier Postlaan 2, Nijmegen 6525 GC, The Netherlands

## Abstract

According to the data from the World Health Organization, acute lower respiratory tract infections, such as pneumonia, are the leading causes of death in low- and middle-income countries, where the burden is highest. Adequate treatment of pneumonia requires accurate diagnosis; however, current diagnostic methods do not allow for rapid differentiation of viral and bacterial cause of pneumonia. Therefore, many patients are empirically treated, which leads to the inappropriate use of antibiotics and the acceleration of the emergence of antimicrobial resistance. We aimed to develop a fast and reliable detection method that is fit for purpose in low-resource settings to identify the most common pathogen causing pneumonia, namely *Streptococcus pneumoniae*. In this study, we developed, optimized, and validated a loop-mediated isothermal amplification (LAMP) method with simplified sample processing to detect *S. pneumoniae* in clinical swab samples. When tested on clinical samples, the simplified LAMP method demonstrated performance to that of the established PCR detection method. However, the LAMP approach does not require an expensive thermal cycler and allows for sensitive detection with a short turnaround time. Moreover, we engineered a simple, affordable device that, together with the method, can be implemented in low- and middle-income countries for point-of-care applications.

## Graphical abstract

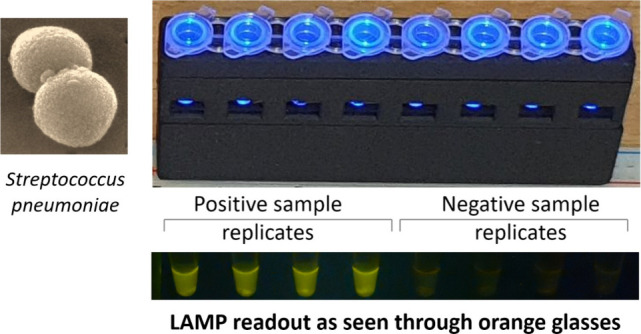

## Introduction

Today, the medical and scientific community is dedicated to preventing the further increase and global spread of antimicrobial resistance and its consequences.^1^ Acute lower respiratory tract infections, such as pneumonia, are leading causes of death in low- and middle-income countries,^2^ particularly in children under 5 years of age. *Streptococcus pneumoniae* and various viruses are responsible for most lower respiratory tract infections.^3–5^ However, accurate epidemiologic data on the causative agents of pneumonia are lacking due to diagnostic limitations, including rapid identification of viral and bacterial causes.^6^ This problem is mainly due to limitations in sampling methods, which rely primarily on upper respiratory tract isolates, including nasal or oropharyngeal swabs or sputum. In addition, countries with the highest burden often lack facilities and equipment for rapid and accurate detection of respiratory pathogens. The more traditional, low-tech, culture-based tests typically take one or more days before a result is known. In some low-resource settings even access to traditional culture-based methods is limited. As a result, many cases are treated empirically with antibiotics, potentially leading to overuse and misuse, contributing to the global spread of antimicrobial resistance.^7^ In addition, antibiotic treatment generally disrupts the microbiota, potentially leading to dysbiosis and increasing the likelihood of developing inflammatory bowel disease and even type 2 diabetes.^8,9^

DNA/RNA-based diagnostics provide highly accurate, rapid, and sensitive detection of pathogenic microorganisms. However, molecular biology methods such as polymerase chain reaction (PCR) are costly due to the equipment and reagents required, and their availability is limited in low- and middle-income countries. The sensitivity of loop-mediated isothermal amplification (LAMP) is comparable to that of PCR, but it is faster and less expensive because it does not require thermal cycling, so any thermal bath will suffice.^10^ In addition to LAMP, there are also other approaches that benefit from the absence of thermal cycling, such as isothermal amplification method - Recombinase Polymerase Amplification (RPA)^11^ or Rolling Circle Amplification (RCA),^12^ that benefit from reduced reaction temperature and low number of primers. However, their use is hampered by high reagent prices or the need for costly phosphorylated and highly purified circular templates. LAMP uses a unique strand-displacing DNA-dependent DNA polymerase (e.g., *Bst* DNA polymerase derived from the large fragment of *Geobacillus stearothermophilus* DNA polymerase I).^13^ This DNA polymerase does not require thermal cycling to melt the DNA double strand. The LAMP uses 4 to 6 primers that bind to adjacent regions of a target gene, resulting in highly specific dumbbell-like amplicons.^14^ Currently, LAMP remains the preferred affordable PCR alternative suitable for point-of-care applications. Several LAMP-based methods have been proposed for the detection of *Streptococcus pneumoniae* in clinical patients with pneumonia symptoms.^15–18^ Unfortunately, all of these approaches still require expensive reagents, microfluidic chip or equipment and some involve time-consuming procedures for sample processing, such as centrifugation and DNA isolation. Therefore, this study proposes a simplified LAMP-based point-of-care method for detection of *S. pneumoniae* that could be used in low-resource settings.

## Materials and Methods

### Pneumococcal Cultivation, Heat Inactivation, and DNA Isolation

*Streptococcus pneumoniae* strain TIGR4 was inoculated onto blood agar plates (Becton Dickinson, USA), grown overnight in an incubator at 37 °C in a 5% CO_2_ atmosphere_,_ and inactivated by heating at 56 °C for 30 min. Colony Forming Units (CFU) were determined by serial dilution on blood agar plates to allow enumeration. Pneumococcal cell suspensions were diluted 10-fold in DNase-free PCR grade water and stored at −20 °C until use. Pneumococcal genomic DNA (gDNA) was purified using the DNeasy Blood and Tissue Kit (Qiagen, Germany). The DNA concentration was determined spectrophotometrically by measuring the absorbance at 260 nm using Nanodrop (ThermoFisher Scientific, USA).

### Clinical Specimen Collection and PCR-Based Detection of *S. pneumoniae*

Swab samples were collected from children aged 2–14 years prior to (adeno)­tonsillectomy using the Copan Universal Transport Media Collection, Preservation, and Transport System (UTM, COPAN, Italy). All swabs were immediately stored at −80 °C, as previously described.^19^

Ethical approval was obtained from the ethics committees of the hospitals involved in the study as described by Katundu et al. (2023), as well as from the National Institute of Medical Research in Tanzania. Written informed consent was provided by the parents or legal guardians of all participating children. The trial was registered with the Pan African Clinical Trials Registry (identifier: PACTR201905466349317).

Quantitative PCR (qPCR) was performed to detect *S. pneumoniae* in the swab samples, as previously described.^4^ Stored swab samples were thawed on ice and vortexed. From each sample, 100 μL was aliquoted into a 96-well plate. The plate was incubated at 93 °C for 15 min to lyse the bacteria. The qPCR was performed using the Biorad CFX96 Touch Real-Time PCR Detection System. All reactions were performed in a final volume of 10 μL containing: 1 μL template, 5 μL SsoAdvanced Universal Probes Supermix (Bio-Rad, USA), 400 nM of each primer and 200 nM probe (Table 1). Each 96-well plate contained negative controls (without DNA template) in duplicate and a seven-step 10-fold serial dilution of the positive control, starting with approximately 10 ng of purified DNA. The qPCR program consisted of a 3 min incubation at 95 °C followed by 50 cycles of 10 s at 95 °C and 20 s at 60 °C. The fluorescence was measured after each cycle. To accurately compare results within targets, the baseline threshold was adjusted so that the positive controls had the same quantification cycle (Cq) value per target for all plates. The Cq cutoff was set at 36 for each target.

**Table 1:** qPCR Primers Targeting *lytA* Gene of *S. pneumoniae*

primer	oligonucleotide DNA sequence
forward	ACGCAATCTAGCAGATGAAGCA
reverse	TCGTGCGTTTTAATTCCAGCT
probe	5′-FAM-GCCGAAAACGCTTGATACAGGGAG-3-BHQ1

### LAMP Primer Design

Primers were designed to target the highly conserved segments of the essential bacterial gene *piaB*. The conserved sequence was identified using sequences from *Streptococcus pneumoniae* strains obtained from public databases. Multiple sequence alignment of 154 DNA sequences found for the piaB gene was used to calculate a consensus DNA sequence using the Tcoffee algorithm.^20^ The most conserved *piaB* segment, based on the genome sequences of strain TIGR4 (GenBank AF338658.1, sequence 1130–2137), was then used for this LAMP assay design using the NEB LAMP Primer Design Tool (https://lamp.neb.com/) (Table S1 in the Supporting Information). Primers were synthesized by Sigma-Aldrich (USA) and included forward outer primer (F3), backward outer primer (B3), forward inner primer (FIP), backward inner primer (BIP), and loop forward (LF) and loop backward (LB) primers (Table 2).

**Table 2:** Selected LAMP Primers Targeting the *piaB* Gene of *S. pneumoniae*

LAMP primer	oligonucleotide DNA sequence
F3	AAATAGCAATCGGACTTGG
B3	GCTGGTAAAATATTTTGAGAGAA
FIP	AAGCTATTGGTCCTGTAATTGAAGTAATCCCGAGCTTTCAAGG
BIP	GGTCCCATAGCCTTAAATATTGGCACAAAACTAGTAAAATTCCAACC
FL	GCAGTTAAAGATACAGCGCAAA
BL	AGCCCAATATTAGCTGGAT

### LAMP Reaction

The analytical sensitivity of the LAMP assay was determined using a 10-fold serial dilution of TIGR4 gDNA (10 ng/μL). The LAMP reaction was carried out in a reaction mixture containing the following reagents: Saphir *Bst* Turbo GreenMaster mix (Jena Bioscience, Germany), 0.2 μM F3 and B3 outer primers, 1.6 μM inner primers (FIP and BIP) and 0.4 μM loop primers (LF and BL) and 45% of the total volume was template solution (purified DNA, bacterial culture or clinical sample). Total reaction volumes were 10, 50, or 25 μL for purified DNA solution, bacterial cultures, or clinical samples, respectively. The samples containing whole bacteria or clinical samples were heat treated at 95 °C for 15 min to inactivate bacteria and facilitate cell lysis (Figure 1 - Step 1). In the case of clinical samples stored in a UTM, the samples were diluted 4 times with distilled water to prevent inhibition of the LAMP reaction (Figure 1 - Step 2). The swab samples stored in the UTM used here did not interfere with our simplified LAMP method.

**Figure 1:**
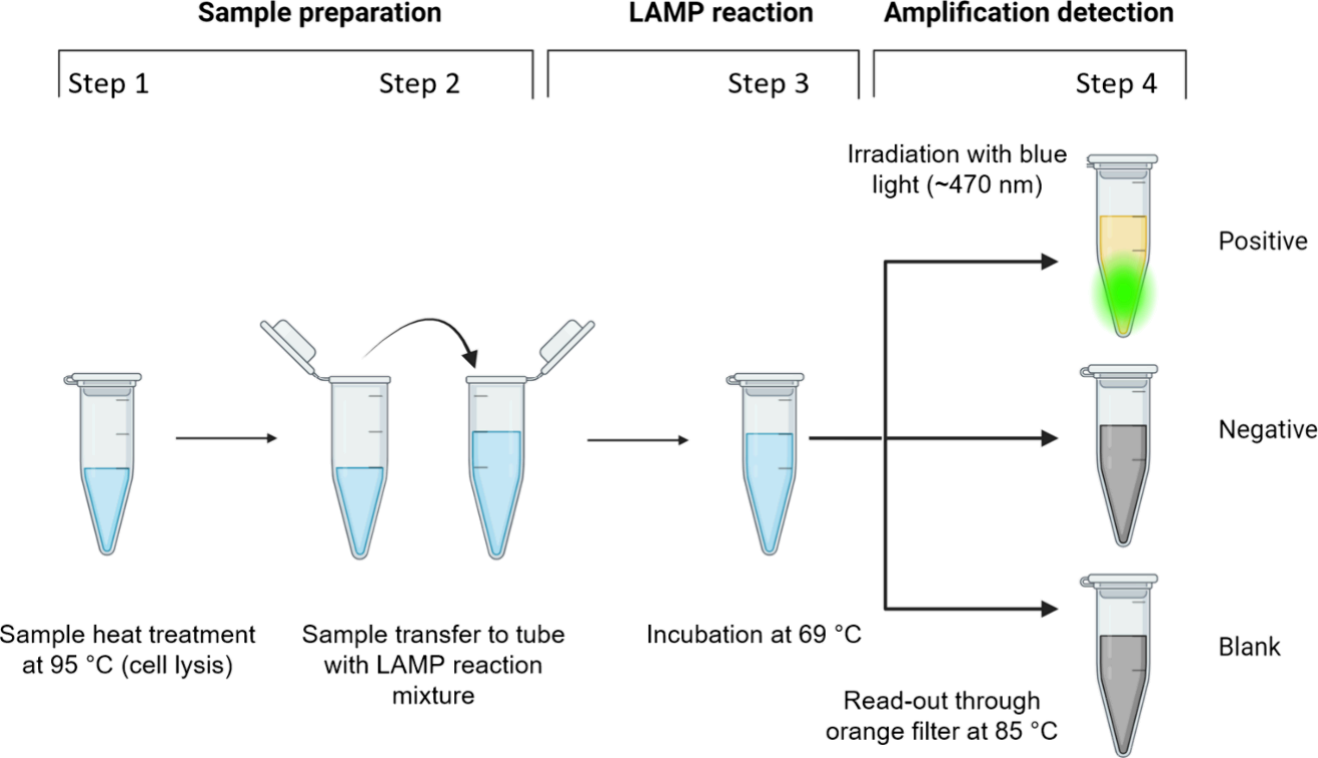
Schematic of a simplified assay procedure for clinical specimens. First, the sample is heated to 95 °C to lyse the bacterial cells step 1), then the sample is transferred to the LAMP reaction mixture (step 2), incubated at 69 °C (step 3), and finally, the fluorescence intensity is detected (step 4).

Amplification was monitored using a Bio-Rad CFX-Connect Real-Time qPCR thermal cycler on the 6-carboxyfluorescein (FAM)/SYBR Green I channel under isothermal conditions (69 °C) (Figure 1 - Step 3). The threshold time was automatically determined for each well by qPCR software (Bio-Rad CFX Maestro 1.1, Bio-Rad, USA) using a built-in nonlinear regression model.

LAMP reading was based on SYBR Green I, which is included in the Saphir *Bst* Turbo GreenMaster Mix (Jena Bioscience, Germany). SYBR Green is an intercalating dye with an excitation wavelength of 495 nm and an emission wavelength of 521 nm when bound to dsDNA. We confirmed that the concentration of DNA produced by the LAMP reaction was sufficient for visual detection using a commercially available transilluminator, Dark Reader Non-UV Transilluminators (Clare Chemical, USA), with an excitation wavelength of approximately 470 nm, in combination with an orange filter (590–625 nm). Prior to reading, LAMP reactions were preheated to 85 °C to reduce the background fluorescence of primers (Figure 1 - Step 4).

We also developed a simplified and low-cost alternative to the commercial transilluminator using 3D-printed components (see below for details), eight blue light LEDs, orange foil/glasses, a generic breadboard, wires, and 2 or 3 AA batteries (number of batteries depends on the type of diodes used).

### 3D Print

We used the Original Prusa MK4S 3D Printer Kit (Prusa Research, Czech Republic) and carbon fiber composite filament XT-CF20 (colorFabb, Netherlands). The print quality was set to “Detail”, and the infill was set to 90% to improve the strength and thermal conductivity of the 3D printed parts.

## Results

### Primer Design and Detection Limit for LAMP-Based *S. pneumoniae* Assay

The LAMP primers used in this study target the *piaB* gene that encodes the permease of the ABC transporter, which is essential for bacterial iron uptake.^21^ This gene was previously found to be a 100% specific target for *S. pneumoniae* and was not detected in common oral streptococci, such as *S. mitis*.^22^

Proposed method performance was tested on three sample types: (i) dilution series of isolated bacterial DNA solutions, where the DNA concentration could be determined spectrophotometrically, (ii) dilution series of cultured pneumococcal cells, where the number of viable cells (CFU) could be determined and (iii) on clinical samples with unknown concentration of the pneumococcal cells. In this third test the performance of newly proposed simplified LAMP was compared to the established method (PCR) in a blind manner.

First, the detection limit for the assay was determined using purified bacterial gDNA isolated from *S. pneumoniae* under the optimal reaction conditions. The LAMP reaction time was found to be linearly proportional to the logarithm of the number of target gDNA up to, but not including the detection limit of 20 copies/μL (see Figure 2). Multiple measurements were performed, and it was concluded that gDNA template concentrations near the detection limit exhibited significant signal variation, with a coefficient of variance of approximately 25%. We noticed that when multiple measurements are performed at concentrations near the detection limit, the results are often inconsistent (some replicates are positive and some are negative). Consequently, we consider these boundary conditions unreliable for pathogen detection when only a single measurement per sample is performed. However, detection sensitivity improves with increasing numbers of replicates per sample. Based on this, we recommend analyzing at least four replicates per sample. This multireplicate approach was subsequently applied to the analysis of both cultured pneumococci and clinical samples.

**Figure 2:**
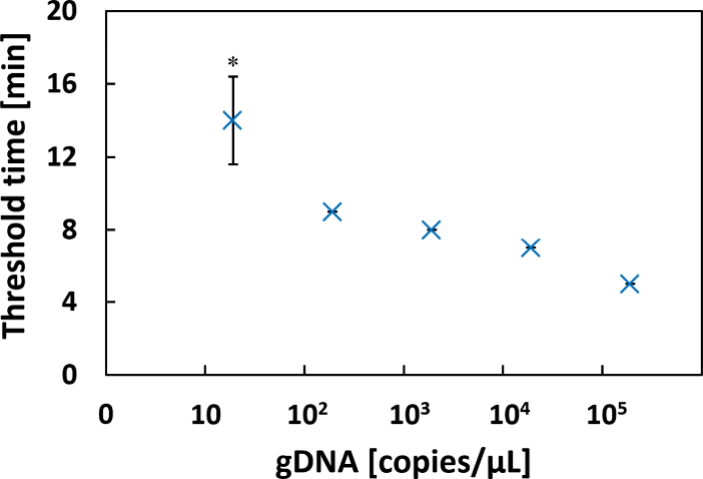
Detection limit (analytical sensitivity) of the LAMP assay. The average LAMP reaction time to reach the threshold is plotted against the concentration of *S. pneumoniae* gDNA (plotted on the *x*-axis using a logarithmic scale). Error bars represent standard deviations from four measurements; the mean and SD for gDNA concentration 20 copies/μL (marked with an asterisk) were calculated from eight measurements. Supplementary Figure S1 shows the time course of the relative fluoresce signal for corresponding LAMP reactions.

### Detection Limit of the LAMP Assay Using Pneumococcal Cells as a Template

The genome of *S. pneumoniae* consists of a single circular DNA molecule,^23^ so in theory the detection limit for gDNA and pneumococcal cells should be similar. In practice, however, other factors, such as DNA availability, come into play. The release of target DNA from cells is a common step prior to the DNA-based detection of intact bacterial pathogens. To simplify sample preparation and eliminate the need for additional instrumentation, we sought to omit this laborious step of DNA extraction or centrifugation typically used to process samples prior to PCR or LAMP analysis. Fortunately, heating the bacterial suspension containing 10^2^ CFU/μL at 95 °C for 15 min was found sufficient to release detectable amounts of DNA from *S. pneumoniae*. Thus, after the heat treatment alone, the detection limit for bacterial samples increased to ∼ 100 bacterial cells/μL (Figure 3). Additionally, in the experiment with freshly cultured pneumococci, low numbers of viable cells (10 CFU/μL) were subjected to eight parallel measurements. Three out of eight reactions were positive (see red dotted curves in Figure 3), indicating that repeated analyses, for which this LAMP setup is highly suitable, could increase the sensitivity of detection. Thus, performing multiple analyses may make this assay more sensitive and reliable when reanalyzing inconclusive results is needed or when trace levels of bacteria are expected.

**Figure 3:**
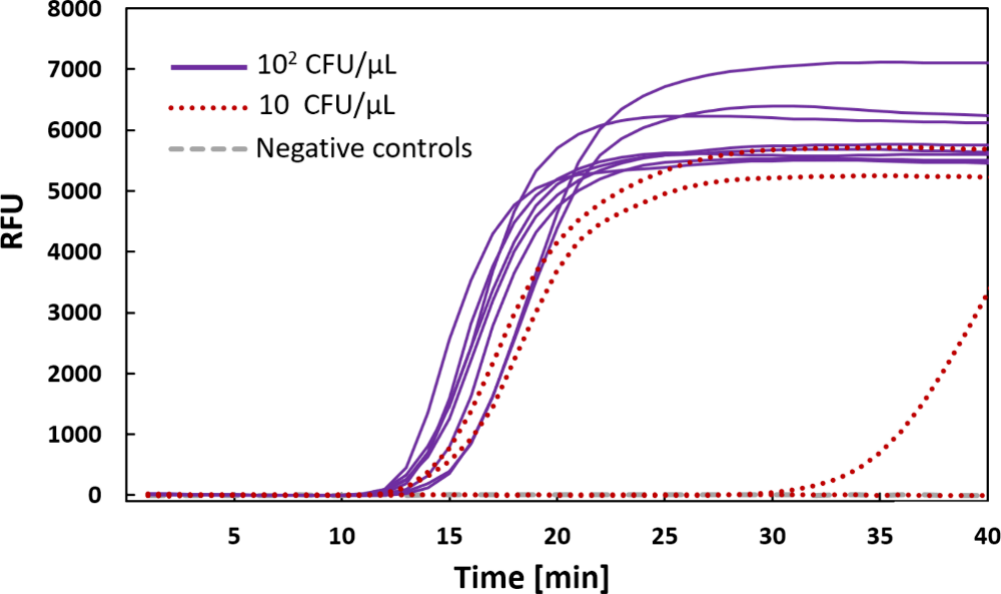
Amplification curves of LAMP assay. Samples contained cultured *S. pneumoniae* bacterial cells diluted to a concentration of 10^2^ CFU/μL (violet solid line), 10 CFU/μL (red dotted line), and negative control (gray dashed line). The graph displays the relative fluorescence units (RFU) of eight replicates for each sample in time (lines showing negligible fluorescence overlap near the zero line).

Although the results indicate that the sensitivity of the simplified LAMP reaction is 5 times lower when performed on whole bacteria compared to isolated DNA, it may still be sufficient for detecting *S. pneumoniae* in clinical samples. We have tested this simplified approach on clinical samples to confirm that it is a viable trade-off for LAMP acceleration and simplification.

### Performance of the Simplified LAMP Assay on Clinical Specimens

The diagnostic sensitivity of the assay was tested by comparing the performance of the simplified LAMP assay with PCR on the same set of clinical isolates. Nasopharyngeal swabs were obtained from patients undergoing adenotonsillectomy, analyzed by a standard PCR method, as previously described,^19,24^ and compared with the simplified LAMP method. The LAMP- and PCR-based read-outs were performed in a blinded manner with four replicates per sample (Table 3).

**Table 3:** Detection of *Streptococcus pneumoniae* in 16 Nasopharyngeal Swabs Using LAMP and PCR Methods

sample #	threshold time of LAMP (min)^a^	% of positive results by LAMP parallels	positive (+)/Negative (**-**) by LAMP	PCR results (Cq)	positive (+)/Negative (**-**) by PCR
1	16 ± 1	100%	+	28	+
2	16 ± 2	100%	+	19	+
3		0%	–		–
4	20 ± 5	100%	+	35	+
5	13 ± 0	100%	+	27	+
6		0%	–	–	–
7	17 ± 0	100%	+	27	+
8		0%	–	–	–
9		0%	–	–	–
10	16 ± 2	100%	+	35	+
11	25	25%	+^b^	36	+
12	32 ± 15	75%	+^b^	36	+
13	14 ± 0	100%	+	30	+
14	18 ± 0	100%	+	27	+
15		0%	–	–	–
16	11 ± 1	100%	+	25	+

a The LAMP reaction was monitored continuously using qPCR for 60 min. Each value represents an average and standard deviation calculated from four measurements.

b In these samples, concentrations of *S. pneumoniae*cells were close to the detection limit for the LAMP method (some replicas out of four parallel samples were positive, and some were negative).

The two clinical samples (#11 and #12) contained low levels of pneumococcal DNA (approaching the detection limit of PCR) because they required the highest number of PCR cycles (more than 35). In addition, these two near-threshold samples only yielded positive results in some LAMP replicates (Table 3), indicating that the LAMP is also approaching its limit of detection for these samples (#11 and #12).

The advantage of the LAMP reaction is that it produces large amounts of DNA in a quick manner, allowing visual detection of the fluorescent end point with a simple transilluminator. However, it is important to find appropriate reading conditions to determine whether the sample is *S. pneumoniae* positive or negative. In particular, it is necessary to maintain the optimal reading temperature to prevent the formation of nonspecific secondary structures of the primers at low temperatures and to prevent the complete melting of the LAMP amplicons at high temperatures. For the present primer design, the optimal prereading warming temperature was determined to be 85 °C. After removing the reaction products from the prewarmed bath and placing them in the fluorescence reader, it is critical to read the fluorescence immediately (within 1 min) because of the substantial increase in background signal (negative control fluorescence) triggered by the formation of short DNA double strands and primer hairpins when the solution temperature drops below approximately 75 °C. Therefore, it is recommended to include at least one negative control sample (without template but with all primers) in each assay to confirm that primer fluorescence is negligible and to ensure that the proper thermal conditions are maintained during the assay.

Commercially available transilluminators are too expensive (500–1,000 USD) to implement in point-of-care applications in low-resource settings. Therefore, we developed and tested a small, low-cost (less than 10 USD) transilluminator device for reading fluorescence in standard PCR strips (Figure 4). This device can be made using a standard 3D printed model (requiring about 15 g of print filament), eight blue light LEDs, wires, AA batteries, and a thermostat that requires electric socket power (e.g., any Mini Dry Bath Incubator) or alternatively a heating bath with a thermometer. The resulting fluorescence can then be read visually with an orange filter, at a total cost of about 0.5 USD per reaction for the LAMP reagents.

**Figure 4:**
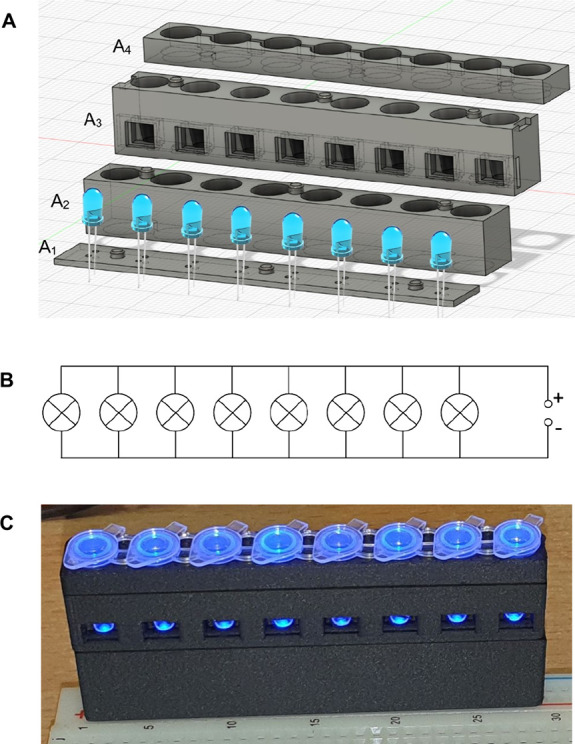
3D model of a compact homemade fluorescence reader or transilluminator (A). The 3D printed holder is assembled from four parts: the LED connection holder (A_1_), the shade segment for 5 mm blue LEDs (A_2_), the sample observation segment (A_3_), and the sample bouncer (A_4_). The schematic of the electrical circuit is shown (B). An example of a system setup using a 3D printed homemade fluorescence reader to read strips containing LAMP reaction products is shown (C). A solderless breadboard can be used for convenient circuit wiring. Three rechargeable AA batteries (3 × 1.2 V) power eight blue LEDs (most major suppliers produce suitable blue LEDs that emit light in the 450 to 475 nm range). An orange filter can be built into the viewing segment of the holder, or more conveniently, orange glasses can be used instead.

The commercial instrument provides more uniform illumination during reading than the homemade instrument. However, the large amount of DNA produced during LAMP amplification results in strong fluorescence signals that are easy to observe even with a simple setup. Positive samples exhibit strong yellow-green fluorescence at 85 °C, while negative samples appear almost dark under the same conditions (see Figure 5). Both commercial and homemade transilluminators can distinguish between positive and negative samples under the proposed conditions. Therefore, homemade transilluminators can be a cost-effective alternative to commercial devices for fluorescence visualization of LAMP products.

**Figure 5:**
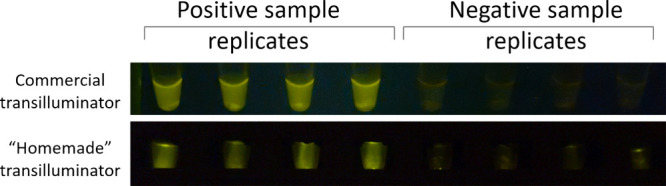
An example of end point fluorescence detection of the LAMP amplification products representing positive and negative clinical samples, corresponding to clinical samples #2 and #3, is shown in Table 3, respectively. Tubes preheated to 85 °C were illuminated with blue light (∼470 nm) and photographed through the orange filter (590–625 nm) using transilluminator Dark Reader (“commercial transilluminator”) or homemade transilluminator from Figure 4.

## Discussion

PCR is a commonly used technique for pathogen detection in clinical laboratories in developed countries. However, there is a growing need for more accessible and less expensive methods suitable for low-tech and resource-poor settings in less developed countries or infrequent providers of basic medical diagnostics. A rapid method that does not require a professional operator and is ideal for point-of-care diagnostics that can be performed away from clinical laboratories would be helpful worldwide. Therefore, there is a growing effort to develop an alternative DNA/RNA-based detection method using isothermal amplification that is much less demanding on instrumentation equipment and has diagnostic potential equivalent to PCR. LAMP-based detection is the most widely used alternative to PCR,^25^ although other isothermal methods are available, including recombinase polymerase amplification (RPA)^26^ and rolling circle amplification (RCA).^27^ However, these approaches are more expensive than LAMP due to patent protection or the requirement to use 5′-end phosphorylated probes.^26,27^

Recently proposed clinical applications using LAMP approaches are rapidly expanding,^28–31^ and some are focused on resource-poor regions. For example, DNA-functionalized gold nanoparticles have been developed that can detect *M. tuberculosis* in sputum samples by visual LAMP with the naked eye.^32^ The strength of this approach is that it requires no instrumentation; however, it does require custom reagents (DNA-functionalized gold nanoparticles) that must be prepared for each LAMP design. On the other hand, our LAMP-based pathogen detection approach using the low-cost fluorescence reader is based on standard oligonucleotide primers and commercially available LAMP kits. We believe the approach proposed here is more convenient to use and can be easily adapted to other primer sets.

A fluorescence reader was the only instrument (besides the temperature-controlled bath) necessary for our LAMP-based approach. To make it more accessible, we developed and tested a simple homemade version of the fluorescence reader (Figure 4) that can be assembled using a common solderless field platform, blue LEDs, and a 3D-printed holder. The best thermoplastic material for this 3D printing application is the carbon fiber-filled filament, which combines very low translucency, sufficient mechanical strength, high thermal conductivity and good heat resistance (tolerating temperatures around 90 °C). This material is not required but is recommended because of its higher thermal resistance. It would allow the transfer of the simplified LAMP method to other pathogens or different primer sets that may require, for example, optimization of the thermal conditions, namely the reaction and detection temperatures.

Nucleic acid template molecules must be made available for amplification during sample processing. This processing often involves cell disintegration and DNA/RNA extraction steps.^33^ Unfortunately, these steps are laborious, time-consuming, and costly, requiring additional time, instrumental equipment, and materials or kits. Among the numerous cell lysis methods available, the thermal lysis used in our proposed method requires a tempered bath (possibly the same one used for LAMP incubation) and does not alter the chemical composition of the sample (thus avoiding possible interference with the LAMP reaction). However, thermal lysis is considered suitable for lysis of cells with weak or no cell walls.^34^ We found that even a brief heat treatment released enough *S. pneumoniae* gDNA allowing LAMP-based pathogen detection in swab samples. This could significantly simplify the sample processing step and eliminate the need for more laborious cell lysis steps such as centrifugation and DNA/RNA isolation. Recently, another extraction-free LAMP method was proposed for rapid and specific detection of wood-decaying fungi showing comparable sensitivity as real-time quantitative PCR without any necessary DNA isolation.^35^

Another optimization step important for developing a reliable LAMP method focuses on the total LAMP reaction volume. It should be noted that the relatively large volume of the biological sample added during the analysis could increase the risk of hampering the polymerization reaction or the detection of DNA products. This is especially true in cases where the samples analyzed were collected from complex matrices containing compounds known to inhibit the polymerase reaction or quenchers that interfere with fluorescence detection. Fortunately, the LAMP reaction we propose for the *S. pneumoniae*detection is sufficiently robust and has been shown to be comparable or even more tolerant to the common inhibitors than a PCR.^36^

The assay described in this study allows rapid identification of *S. pneumoniae* in clinical samples without the need for complicated or expensive instrumentation. The assay typically took 30 min (up to 50 min) including sample processing. However, the simplification comes at the cost of a lower sensitivity compared to the same method using purified DNA as a template. The other works, which focused on different clinical sample types (cerebrospinal fluid or sputum samples processed by alkaline DNA extraction), reported their LAMP sensitivity for detecting pneumococci in the range of units and tens of copies/μL.^16,17^ This range is comparable to the detection limit of 20 gDNA copies/μL reported here for isolated *S. pneumoniae* DNA. If the DNA isolation step is replaced with a short heat treatment, as in our simplified LAMP preparation protocol, the sensitivity for detecting cultured *S. pneumoniae* cells drops to ∼ 100 copies/μL. Although the simplified LAMP method has the disadvantage of slightly lower sensitivity as a trade-off for rapid sample preprocessing, it performed well on clinical samples when compared to classical PCR. While the previously described LAMP methods using full sample preprocessing steps have been reported to be more sensitive than PCR,^37^ some of them are able to detect DNA templates approaching the single molecule level.^38^ However, such high sensitivity can be a complication for accurate clinical diagnosis, as oversensitive methods are (i) highly susceptible to carryover contamination, resulting in more frequent false positives, and (ii) it may also detect low numbers of pneumococci in individuals with very low bacterial loads (pneumococcal carriers), where the presence of bacteria is unrelated to the ongoing disease. Therefore, the level of sensitivity of the simplified LAMP described here may improve clinical diagnostics, as it prevents the problem of false-positive detection of pathogens in oro/nasopharyngeal swab material associated with very high qPCR sensitivity.^39^

## Conclusions

Here, we proposed to combine the advantages of high sensitivity and specificity of fluorescent LAMP detection with a low-cost read-out method. To analyze clinical samples containing intact *S. pneumoniae* cells, centrifugation, disintegration and DNA extraction steps could be omitted and replaced by a short heat treatment. The simplified LAMP method shows a detection limit of up to 100 bacterial cells/μL. The performance of the simplified method on clinical samples is comparable to the classical PCR-based detection. The sensitivity could potentially be further increased by performing the test in multiplicates.

Fluorescence visualization of the LAMP product could be performed using a simple and inexpensive homemade device at a total cost of approximately 0.5 USD per reaction. No tube opening is required (and not recommended) after amplification, thus minimizing the risk of carryover contamination. Therefore, we propose a substantially improved, rapid, reliable, and sufficiently sensitive LAMP method suitable for detecting *S. pneumoniae*in clinical samples. As the proposed method has been successfully tested on isolated bacterial DNA, intact cultured pathogen cells, and clinical swab samples, we expect it to perform well on other types of clinical isolates. However, this should be confirmed in further studies.

## Supplementary Material



## Data Availability

The file containing the.stl models for 3D printing of the homemade fluorescence reader is available under Creative Commons license (*CC BY-NC-SA* 4.0) on www.printables.com https://www.printables.com/model/1336591-homemade-fluorescence-reader-for-pcr-8-strip-tubes

## Notes

Parameters used in the NEB LAMP Primer Design Tool to design primers targeting the piaB gene of *Streptococcus pneumoniae*; amplification curves of the LAMP assay (PDF)

## Abbreviations

CFU: colony forming units
LAMP: loop-mediated isothermal amplification
PCR: polymerase chain reaction
UTM: universal transport medium
