# Supplementary material for: Simplified Loop-Mediated Isothermal Amplification-Based Method for Point-of-Care Detection of Streptococcus pneumoniae in Low-Resource Settings
Source: ACS Omega. 2025 Jul 9;10(28):30250–8. doi: 10.1021/acsomega.5c01541 (PMC12290675; doi:10.1021/acsomega.5c01541)
Supplement: Supplementary file 1 [file ao5c01541_si_001.pdf]

Supplementary information

Simplified LAMP-based method for point-of-care detection of Streptococcus pneumoniae in low-resource settings

Petr Jeřábek[1], Markéta Martínková[1], Matúš Friček[1], Christa E. van der Gaast – de Jongh[2], Denis R. Katundu[3,4], Niels van Heerbeek[3,4], Corné H. van den Kieboom[5], Marien I. de Jonge[2]*, Václav Martínek[1]*

[1] Department of Biochemistry, Faculty of Science, Charles University, Prague, The Czech Republic

[2] Department of Laboratory Medicine, Laboratory of Medical Immunology, Radboud Center for Infectious Diseases, Radboud University Medical Center, Nijmegen, The Netherlands

[3] Department of Otolaryngology, Kilimanjaro Christian Medical University College, Kilimanjaro, Tanzania

[4] Department of Otolaryngology, Head and Neck Surgery, Radboud University Medical Center, Nijmegen, The Netherlands

[5] Xheal B.V., Reinier Postlaan 2, 6525 GC, Nijmegen, The Netherlands

*Corresponding authors. E-mail: vaclav.martinek@natur.cuni.cz, Marien.DeJonge@radboudumc.nl

Table S1. Parameters used in the NEB LAMP Primer Design Tool to design primers targeting the *piaB* gene of *Streptococcus pneumoniae*.

**General**	Sorting Rule	Easy
**Reaction Conditions**	Na^+ concentration	10.0 mM
	Mg^{2+} concentration	8.0 mM
**Lengths (min/max)**	F1c/B1c	20 nt – 25 nt
	F2/B2	18 nt – 25 nt
	F3/B3	18 nt – 25 nt
	LF/LB	13 nt – 25 nt
**T_m (min/max)**	F1c/B1c	60 ˚C – 63 ˚C
	F2/B2	55 ˚C – 58 ˚C
	F3/B3	55 ˚C – 58 ˚C
	LF/LB	55 ˚C – 66 ˚C
**% GC**	Min/Max	30 % – 65 %
	Min/Max (Loop)	40 % – 65 %
**ΔG threshold**	5′ Stability	-3 kcal/mol
	3′ Stability	-4 kcal/mol
	3′ Stability (Loop)	-2.0 kcal/mol
	Dimer check	-2.5 kcal/mol
	Dimer check (Loop)	-3.5 kcal/mol
**Distances (min/max)**	(F2-B2)	120 nt – 180 nt
	Loop (F1c-F2)	40 nt – 60 nt
	F2-F3	0 nt – 20 nt
	F1c-B1c	0 nt – 100 nt
**Limits***	F1c/B1c	3
	F2/B2	10
	F3/B3	3
	LF/LB	10
	Sets	1000

nt = nucleotides

* maximum number of combinations during primer set generation

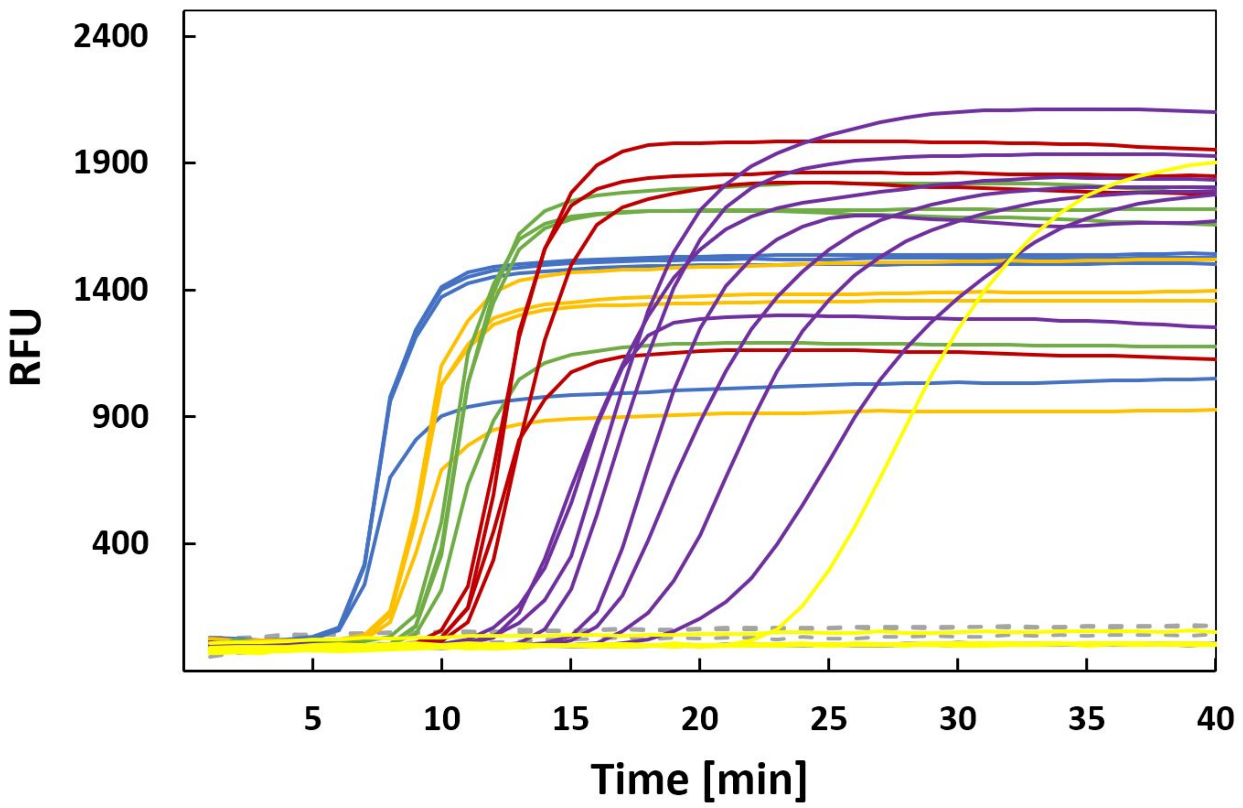

Figure S1. Amplification curves of the LAMP assay. Samples containing various concentrations of gDNA: 5×10^5 (blue), 2×10^4 (orange), 2×10^3 (green), 2×10^2 (red), 2×10^1 (violet), 2 (yellow), 0 (grey dashed line; negative control) gDNA copies/µL. Note: Lines with negligible fluorescence overlap near the zero line.
